# 17‐β‐estradiol reduces surface PD‐L1 expression in estrogen receptor‐positive breast cancer but not type 1 endometrial cancer cells

**DOI:** 10.1002/ctm2.1330

**Published:** 2023-07-13

**Authors:** Hongzhuo Li, Wenyi Gu, Penny Jeffery, Chen Chen

**Affiliations:** ^1^ School of Biomedical Sciences Faculty of Medicine The University of Queensland Brisbane Queensland Australia; ^2^ Australian Institute for Bioengineering and Nanotechnology The University of Queensland Brisbane Australia; ^3^ School of Biomedical Sciences at the Translational Research Institute Queensland University of Technology Brisbane Queensland Australia

Dear Editor,

We report here that 17‐β‐estradiol (E2), through ERα (estrogen receptor α), inhibits IFN‐γ‐induced surface PD‐L1 (programmed death‐ligand 1, CD274) level in advanced ER^+^/HER2^−^ breast cancer (BC) but not in triple negative BC (TNBC) or endometrial cancer (EC). This study implies that mTOR and MAPK pathways can reduce the surface PD‐L1 level and diminish immune evasion via ERα. It also has important implications on endocrine resistance and the limitations of mTOR or MAPK‐targeted therapies for treating advanced ER^+^/HER2^–^ BC.

Tumour cell surface PD‐L1 binds to the PD‐1 (programmed death 1, CD279) receptor on the surface of activated tumour‐infiltrating lymphocytes (TILs), leading to tumour immune evasion. However, high‐level surface PD‐L1 is observed in ER^−^ BC and EC tissues and the TILs^1^, ^2^ but rarely in ER^+^ BC tumors.^2^ Additionally, it remains unclear why ER^+^ patients are less responsive than those with ER^−^ cancers to anti‐PD‐1/PD‐L1 therapy.

E2 functions primarily through the two receptors of ERα and ERβ in both cancers. Approximately 60%–65% of BC cases are ER^+^/HER2^–^ luminal A subtype,^3^ and 80% of EC are of type 1 ER^+^ cancers.^4^ E2 reduces the IRF‐1 protein and mRNA levels in murine splenocytes^5^ and MCF‐7 cell line,^6^ respectively, implying that E2 may reduce the surface PD‐L1 in both diseases. However, how E2 regulates tumour cell surface PD‐L1 expression in ER^+^ BC and type1 EC is elusive.

By qRT‐PCR, BC cell lines MCF‐7 (ERα^+^/PD‐L1^trace^) and MDA‐MB‐231 (ERα^−^/PD‐L1^high^), and EC cell lines Ishikawa (ERα^+^/PD‐L1^trace^, type 1) and TEN (ERα^−^/PD‐L1^trace^, type 2) were selected; flow cytometry was employed for this study. We examined the PD‐L1 transcripts in a cohort of 25 EC specimens and detected a significantly higher PD‐L1 transcriptional level in the type 2 EC group than in non‐EC, but not for type 1 EC (Tables S1–S4 and Figures S1 and S2).

Since IFN‐γ is a major driver of surface PD‐L1 expression through the IFN‐γ‐JAK‐STAT‐IRF1 pathway in multiple cancers,^7^ we exposed these cells to different concentrations of IFN‐γ for 24 h and demonstrated that IFN‐γ strongly increased the surface PD‐L1 level in MCF‐7, MDA‐MB‐231 and TEN. This did not happen to Ishikawa (Figure 1A–D), even after 3 days of exposure to 50 ng/ml IFN‐γ (Figure 1G,H). Comparatively, MCF‐7 showed a strong response, with the surface PD‐L1 level peaking at 48 h and declining non‐significantly at 72 h (Figure 1E,F). The same trend occurred after the cells were exposed to 10 ng/ml IFN‐γ in a phenol‐red free medium containing 5% CSFBS (charcoal‐stripped fetal bovine serum) for 3 days (Figure 1I,J).

**FIGURE 1:**
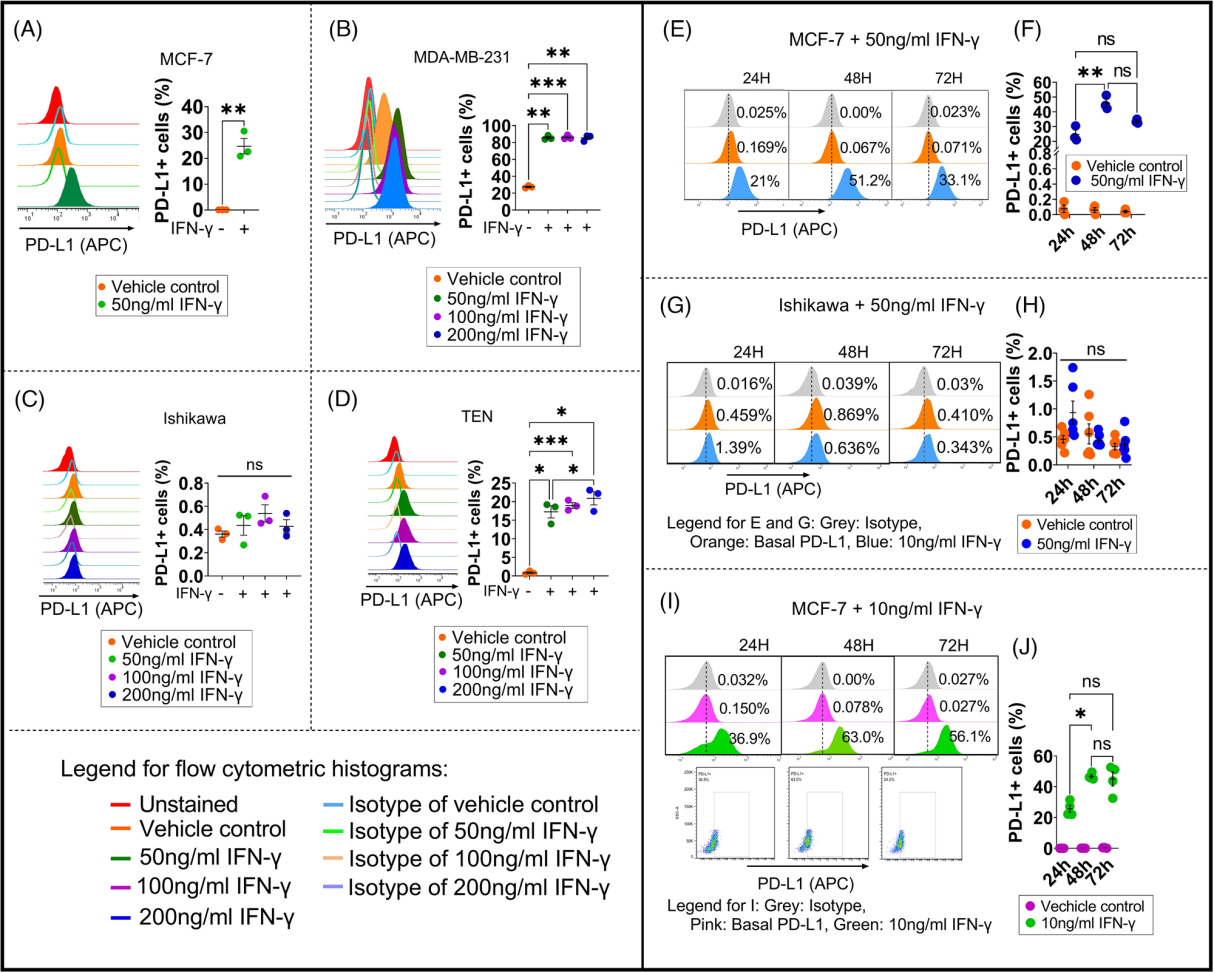
IFN‐γ’s effect on surface PD‐L1 expression in MCF‐7 (A, E, F, I and J), MDA‐MB‐231 (B), Ishikawa (C, G and H) and TEN (D) for indicated times. The surface PD‐L1 levels were assessed by flow cytometry in both BC and EC cell lines exposed or not exposed to different concentrations of human IFN‐γ recombinant protein at indicated time points. Non‐filled (open) peaks and grey peaks: The cancer cells exposed or not exposed to IFN‐γ were stained with the APC‐conjugated mouse IgG1K isotype control antibodies. Orange and pink peaks: The non‐IFN‐γ exposed cancer cells were labeled with APC‐conjugated anti‐human CD274 antibodies. Filled blue, purple and green peaks: The cells exposed to IFN‐γ were labeled with the APC‐conjugated anti‐human CD274 antibodies. (A–D) Left panel: Representative overlay histograms show the surface PD‐L1 levels in MCF‐7 (A), MDA‐MB‐231 (B), Ishikawa (C) and TEN (D) exposed to IFN‐γ (50, 100 and 200 ng/ml) or vehicle control for 24 hours. Under the same condition, the changes in the proportion (%) of the surface PD‐L1^+^ cells in each cell line were compared in the Right panel of A–D. Data are presented as mean ± SEM. Unpaired t‐test (A) or one‐way ANOVA (B–D) was employed with multiple comparisons of Tukey for statistical analyses. (*n* = 3). (E–J) Time course (24–72 h) effect of IFN‐γ (10 or 50 ng/ml) on the surface PD‐L1 expression in Ishikawa and MCF‐7 cells. The cells were grown in either regular (E‐F, MCF‐7, *n* = 3; G‐H, Ishikawa, *n* = 6) or phenol‐red free media (I‐J, MCF‐7, *n* = 4) containing 5% CSFBS. (E), (G), and the upper panel of (I): Representative overlay histograms show the surface PD‐L1 levels in both Ishikawa and MCF‐7 exposed or not exposed to IFN‐γ for 3 days. The lower panel of (I): Representative dot plots display the frequencies of the surface PD‐L1 expressing cells (PD‐L1^+^ cells). The numbers denote the percentages of the gated surface PD‐L1^+^ population in total live cells. Data are presented as mean ± SEM. Two‐way ANOVA was employed with multiple comparisons of Tukey for the analyses of statistics. *p ≤ 0.05; **p ≤ 0.01 and ***p ≤ 0.001. ns: not significant.

We exposed the four cell lines to 10 ng/ml IFN‐γ, 10 nM E2, and a combination of both in a phenol‐red free medium containing 5% CSFBS during an eight‐day culture. We showed that E2 alone did not alter the PD‐L1 level on the surface of all four cell lines but greatly downregulated the IFN‐γ‐induced PD‐L1 at the surface and transcriptional levels in MCF‐7 co‐exposed to E2 and IFN‐γ, compared with IFN‐γ exposure alone (Figure 2A–F), suggesting that it is not endogenous but the IFN‐γ‐induced surface PD‐L1 that was downregulated by E2 in the advanced ER^+^/HER2^−^ BC in vitro.

**FIGURE 2:**
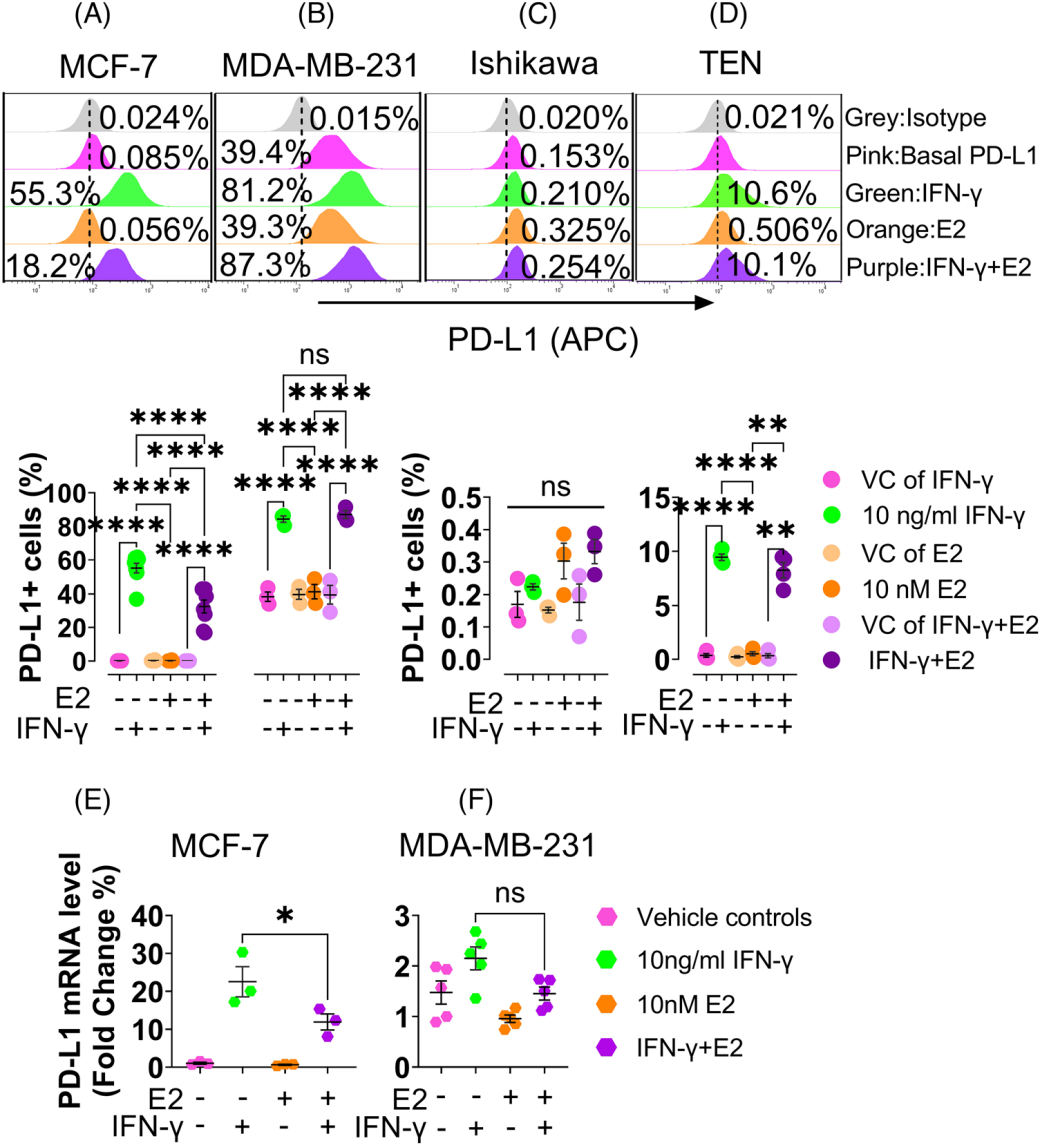
E2's effect on IFN‐γ‐induced surface PD‐L1 expressions in the cell lines of MCF‐7, MDA‐MB‐231, Ishikawa and TEN. Three days after seeding, 10 nM E2 was added to the four cell lines maintained in a phenol‐red‐free medium containing 5% CSFBS. 10 ng/ml IFN‐γ either alone or combined with 10 nM E2 was applied to the cells on day six. 48 hours later (on the eighth day), the above cells exposed to E2, IFN‐γ, or E2 plus IFN‐γ and the vehicle controls were harvested for flow cytometric analysis or qRT‐PCR to determine surface PD‐L1 or PD‐L1 mRNA levels. Pure ethanol and solvent of IFN‐γ (PBS, pH 7.2 plus 0.5% BSA) were used as the negative (vehicle) controls. (A–D) Upper panel: Representative overlay histograms of the surface PD‐L1 expression in each cell line exposed or not exposed to E2, IFN‐γ or the combination of both. Lower panel: Statistical assays of the PD‐L1 at the cell surface levels in the MCF‐7 (A, *n* = 8), MDA‐MB‐231 (B, *n* = 3), Ishikawa (C, *n* = 3) and TEN (D, *n* = 4) cells. (E and F) The IFN‐γ‐induced PD‐L1 mRNA level was diminished by E2 in the MCF‐7 cells (*n* = 3, the exact p‐value of IFN‐γ (10 ng/ml) + 10 nM E2 vs IFN‐γ (10 ng/ml): *p* = 0.0421) but not in the MDA‐MB‐231 cells (*n* = 5, the exact p‐value of IFN‐γ (10 ng/ml) + 10 nM E2 vs IFN‐γ (10 ng/ml): *p* = 0.2843). Data are presented as mean ± SEM. One‐way ANOVA was employed with multiple comparisons of Tukey for statistical analyses. *p ≤ 0.05; **p ≤ 0.01 and ****p ≤ 0.0001. ns: not significant.

As E2 affected ERα^+^/ERβ^+^ MCF‐7 instead of the ERα^−^/ERβ^+^ MDA‐MB‐231, we used two ERα antagonists tamoxifen (Figure 3A) and ICI 182,780 (Fulvestrant) (Figure 3B), and the ERβ antagonist PHTPP (Figure 3C) to block both receptors, respectively, in MCF‐7. We demonstrated that ERα contributed to the E2's downregulation at the surface and transcriptional (Figure 3D) levels. Since the PD‐L1 level in the ERα^−^/ERβ^+^ A549 lung cancer cell line is strongly upregulated by IFN‐γ via the JAK/STAT/IRF‐1 pathway,^8^ our outcome was further validated in this line that ERβ was not involved (Figure 3E).

**FIGURE 3:**
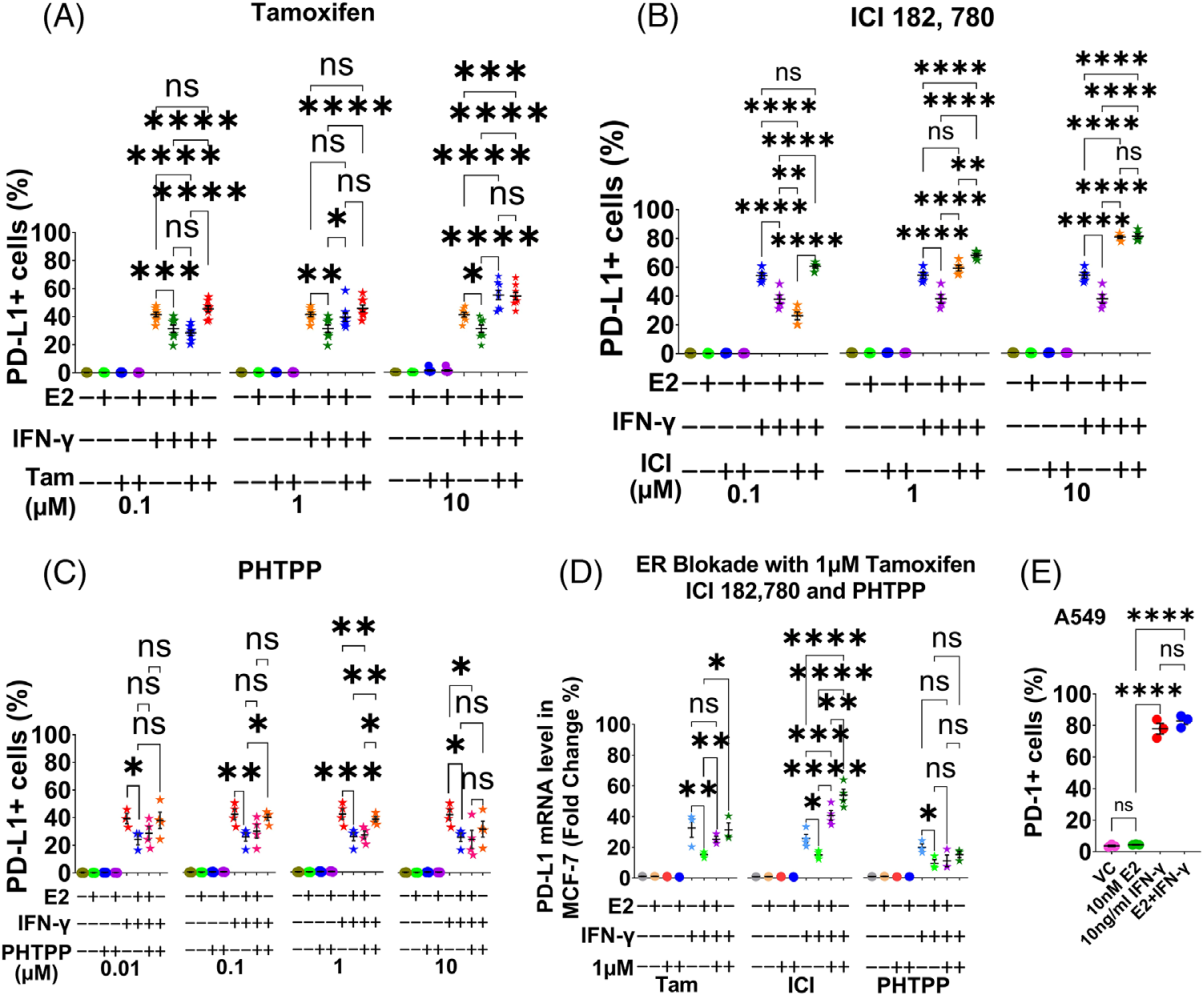
Role of ERα in E2‐downregulated‐IFN‐γ‐induced surface PD‐L1 level in MCF‐7. On the second day after seeding the MCF‐7 cells in a phenol‐red‐free medium containing 5% CSFBS, 10 nM E2 and different concentrations of ER antagonists were added, either alone or in combination. On the third day, 10 ng/ml IFN‐γ was added. 48 hours later, the cells exposed to E2, IFN‐γ, ERα antagonists of tamoxifen (*n* = 8) or ICI 182,780 (*n* = 5), or ERβ antagonist of PHTPP (*n* = 3), either alone or in a combination, along with the corresponding vehicle controls were harvested for flow cytometric analysis or qRT‐PCR to determine surface PD‐L1 or PD‐L1 mRNA levels. (A–C) Statistical analyses of the changes in the surface PD‐L1 expression induced by IFN‐γ following the blockade of ERs with different antagonists. (D) The E2‐downregulated‐IFN‐γ‐induced PD‐L1 mRNA levels in the MCF‐7 cells were determined by qRT‐PCR after the blockade of ERs with 1 μM tamoxifen (*n* = 3), ICI 182, 780 (*n* = 4) and PHTPP (*n* = 3), respectively. (E) The A549 cells were exposed to E2 and IFN‐γ either alone or in combination for 48 h under the five‐day culture condition, proving that ERβ does not influence E2's reducing effect (*n* = 3). Data are presented as mean ± SEM. One‐way ANOVA was employed with multiple comparisons of Tukey for statistics. *p ≤ 0.05; **p ≤ 0.01; ***p ≤ 0.001 and ****p ≤ 0.0001. ns: not significant.

Our finding accords with a newly published work by Hühn et al.,^9^ who showed that total E2 deprivation or fulvestrant treatment increased the surface PD‐L1 expression in MCF‐7 through ERα. Their report designated changes in MFI, however, we observed changes in the proportion (%) of the PD‐L1 expressing cells. This suggests that E2 may protect ER^+^/HER2^–^ BC by interacting with ERα and reducing the quantity of IFN‐γ‐induced surface PD‐L1 expressing cells. Thus, such a protective role by E2 may slow the progression of ER^+^ BC's deterioration. This may explain why ER^+^/HER2^–^ BC expresses less PD‐L1, less malignance than the basal TNBC subtype, and less sensitivity to anti‐PD‐1/PD‐L1 therapy than ER^−^ BC.

We confirmed that the JAK/STAT/IRF1 pathway regulates the IFN‐γ‐induced surface PD‐L1 level (Figure 4,1 and 2A). Co‐exposure to BEZ235 or U0126 combined with E2 and IFN‐γ significantly restored the proportion of the E2‐downregulated IFN‐γ‐induced surface PD‐L1 expressing cells in MCF‐7 (Figure 4,2C), but LY294002 or other kinase inhibitors (Table S5) were not capable (Figure 4,2B,C). Additionally, BEZ235 performed at a much greater recovery rate than U0126, significantly higher than those exposed to IFN‐γ in MCF‐7 (Figure 4,2D). This outcome implies that both the mTOR and the MAPK pathways cooperate in inhibiting immune evasion through ERα by reducing the IFN‐γ‐induced surface PD‐L1 expression in ER^+^ BC.

**FIGURE 4:**
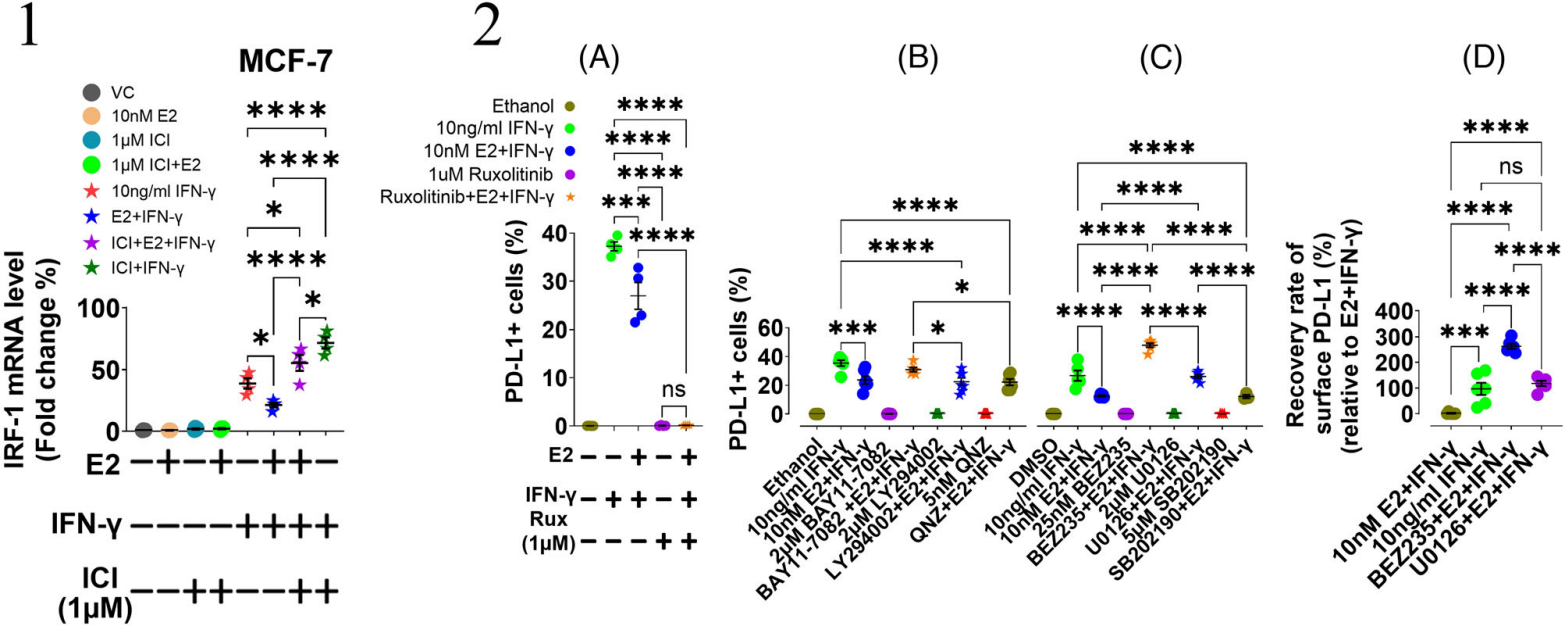
(1) Effect of E2 on IRF‐1 mRNA level induced by IFN‐γ in MCF‐7. The MCF‐7 cells were exposed to E2, IFN‐γ and ICI 182,780 either alone or in a combination for 48 h under the five‐day culture condition. Total RNAs were isolated from each condition and the IRF‐1 mRNA level was examined by qRT‐PCR. The fold‐change in IRF‐1 transcriptional level was shown on the Y‐axis after normalization with the internal control of the GAPDH gene, and relative to the MCF‐7 group without treatment. Data are presented as mean ± SEM. One‐way ANOVA was employed with multiple comparisons of Tukey for statistics. (*n* = 4). *p ≤ 0.05 and ****p ≤ 0.0001. VC: vehicle control. (2) The effects from multiple kinase inhibitors on the E2‐downregulated‐IFN‐γ‐induced surface PD‐L1 expression in MCF‐7. The MCF‐7 cells were cultured under the five‐day culture condition. The cells were treated for one hour with various kinase inhibitors before adding E2 (10 nM) on day two and before adding IFN‐γ (10 ng/ml) on day three, respectively. The proportion (%) of PD‐L1^+^ cells at the surface level under each inhibitory condition was studied by flow cytometry. The restorative rate of the E2‐downregulated‐IFN‐γ‐induced surface PD‐L1 was calculated as Recovery rate = [[(Inhibitor+E2+IFN‐γ)‐(E2+IFN‐γ)] / (E2+IFN‐γ)]*100%. (A) The proportion (%) of the E2‐downregulated‐IFN‐γ‐induced surface PD‐L1^+^ cells was not increased by inhibiting the JAK/STAT pathway with ruxolitinib. (Orange stars of Ruxolitinib + E2 + IFN‐γ vs blue dots of E2 + IFN‐γ, *n* = 4). (B and C) Inhibitors dissolved in ethanol (B) or DMSO (C) were used to inhibit corresponding pathways. (B) The inhibition of the PI3K/Akt pathway by LY294002 did not restore the proportion (%) of the E2‐downregulated‐IFN‐γ‐induced surface PD‐L1^+^ cells (Blue stars of LY294002 + E2 + IFN‐γ vs blue dots of E2 + IFN‐γ, *n* = 6). (C) Significant increases in the proportion (%) of the E2‐downregulated‐IFN‐γ‐induced surface PD‐L1^+^ cells were observed after the PI3K/Akt/mTOR pathway was inhibited with the dual inhibitor BEZ235 (Orange stars of BEZ235 + E2 + IFN‐γ vs blue dots of E2 + IFN‐γ, *n* = 6), while the MAPK pathway was inhibited with the MEK inhibitor U0126 (Blue stars of U0126 + E2 + IFN‐γ versus blue dots of E2 + IFN‐γ, *n* = 6). (D) Comparative analysis of the restorative rate between the mTOR and the MAPK pathways on the E2‐downregulated‐IFN‐γ‐induced surface PD‐L1 expression in MCF‐7. The mTOR pathway has stronger restorative effects than the MAPK pathway. Data are presented as mean ± SEM. One‐way ANOVA was employed with multiple comparisons of Tukey for statistical analyses. *p ≤0.05; ***p ≤ 0.001 and ****p ≤ 0.0001. ns: not significant. VC: vehicle control.

We found that tamoxifen or ICI 182,780 potentiates IFN‐γ to upregulate surface PD‐L1 expression, even when E2 is present in MCF‐7 (Figure 3A,B). The higher the antiestrogen dose, the greater the PD‐L1 surface expression (Figure 3A,B) as ICI 182, 780 inhibited ER in a dose‐dependent manner.^10^ This indicates that prolonged administration of antiestrogens may induce tumour cell surface PD‐L1 expression by disrupting a dynamic protective effect built by the E2‐ERα‐IFN‐γ‐mTOR‐MAPK axis in the tumour milieu of the advanced ER^+^/HER2^–^ BC patients, triggering tumour immune evasion. Together, these findings may provide causal reasons for antiestrogen resistance and the limited efficacy of mTOR or MAPK‐targeted therapies for treating advanced ER^+^/HER2^–^ BC patients. Additionally, the increased tumour cell surface PD‐L1 induced by the inhibitors of ERα, mTOR or MAPK may potentially augment the sensitivity of ER^+^ BC cells to immune checkpoint inhibitors.

## 1 CONCLUSION

E2 reducing the IFN‐γ‐induced cancer cell surface PD‐L1 may be the main reason why high‐level PD‐L1 protein in ER^+^ BC tumours is rare. Antiestrogens or targeted therapies combined with anti‐PD‐1/PD‐L1 regiments could be more beneficial in treating advanced ER^+^/HER2^–^ BC patients.

## CONFLICT OF INTEREST STATEMENT

The authors declare no conflict of interest.

## Supporting information

Supporting Information

Supporting Information

Supporting Information
