# Supplementary figures and images for: 17‐β‐estradiol reduces surface PD‐L1 expression in estrogen receptor‐positive breast cancer but not type 1 endometrial cancer cells
Source: Clin Transl Med. 2023 Jul 13;13(7):e1330. doi: 10.1002/ctm2.1330 (PMC10345460; doi:10.1002/ctm2.1330)

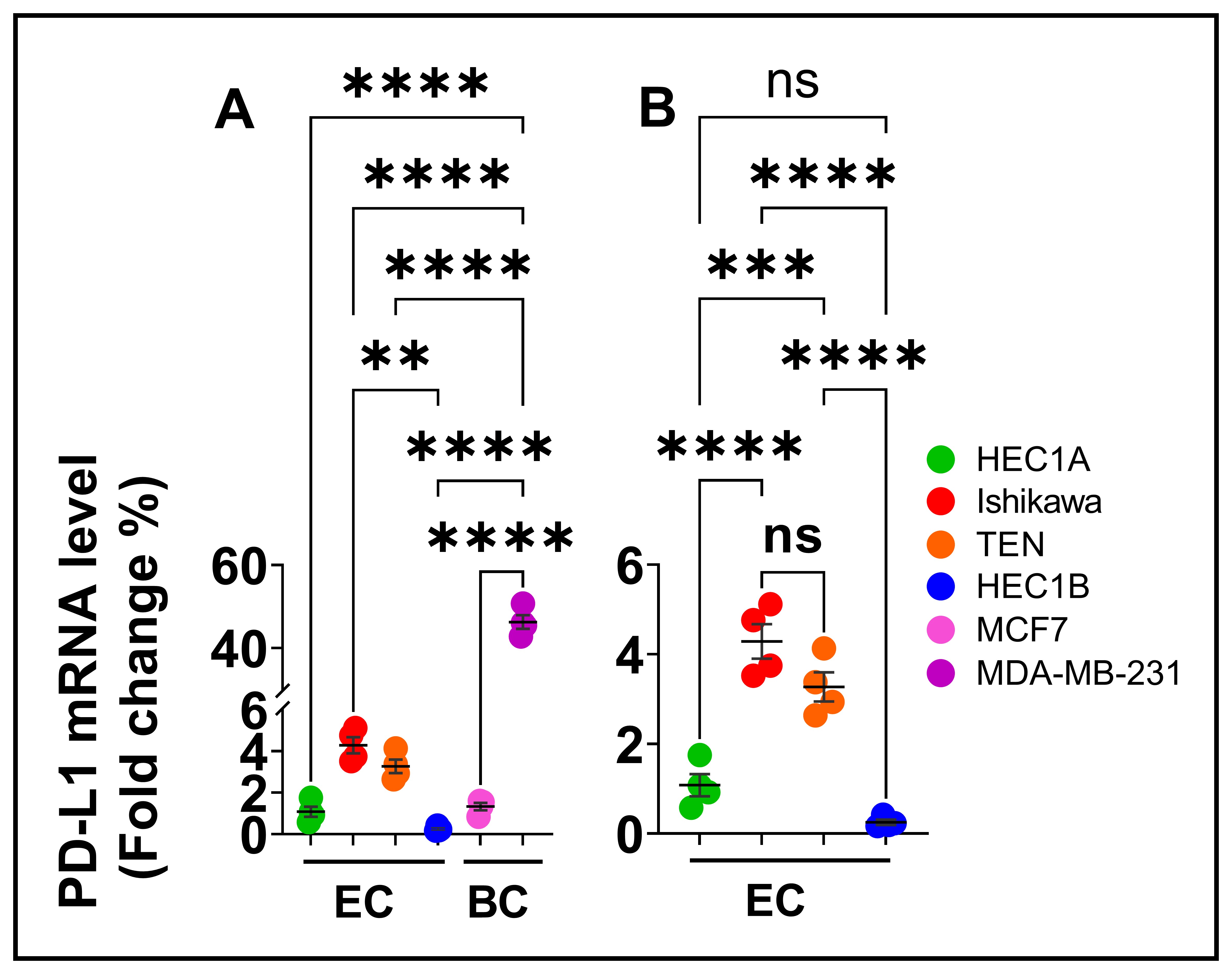

Supplement: Supplementary file 1 — Supporting Information [file CTM2-13-e1330-s002.jpeg]

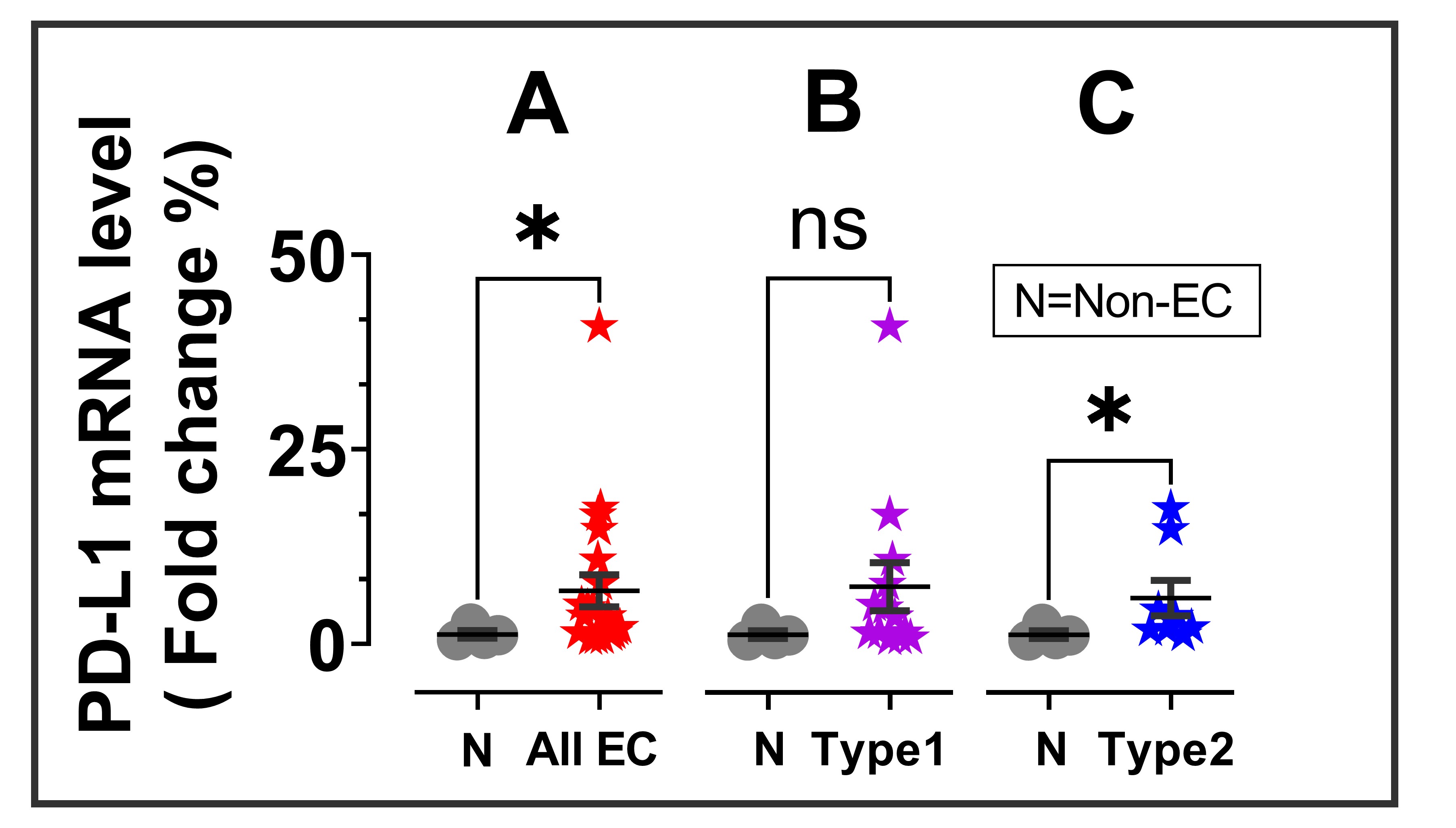

Supplement: Supplementary file 2 — Supporting Information [file CTM2-13-e1330-s001.jpeg]
