# Supplementary material for: 17‐β‐estradiol reduces surface PD‐L1 expression in estrogen receptor‐positive breast cancer but not type 1 endometrial cancer cells
Source: Clin Transl Med. 2023 Jul 13;13(7):e1330. doi: 10.1002/ctm2.1330 (PMC10345460; doi:10.1002/ctm2.1330)
Supplement: Supplementary file 3 — Supporting Information [file CTM2-13-e1330-s003.docx]

**Supplemental Tables:**

Table S1. The primers used in this study

| Genes | **Forward Primers** | **Reverse Primers** |
| --- | --- | --- |
| ***CD274*** | 5’- AGGGCATTCCAGAAAGATGAG-3’ | 5’-TTGGGAACCGTGACAGTAAAT-3’ |
| ***ESR1*** | 5’- GTGAAGCTTCGATGATGGGC-3’ | 5’-CAAATCCACAAAGCCTGGCA-3’ |
| ***ESR2*** | 5’-TCCATCGCCAGTTATCACATCT-3’ | 5’-CTGGACCAGTAACAGGGCTG-3’ |
| ***IRF-1*** | 5’-CATGCCCTCCACCTCTGAAG-3’ | 5’-CCATCCACGTTTGTTGGCTG-3’ |
| ***GAPDH*** | 5’- GACCACTTTGTCAAGCTCATTTC-3’ | 5’-CTCTCTTCCTCTTGTGCTCTTG-3’ |

Note: Corresponding gene names of each protein: PD-L1: *CD274*; ERα: *ESR1*; ERβ: *ESR2*;

IRF-1: *IRF-1* and GAPDH: *GAPDH*

**Table S2. *ESR1* and *ESR2* mRNA expressions in BC and EC cells**

| Cell LinesESRs | BC cell lines | | EC cell lines | | | |
| --- | --- | --- | --- | --- | --- | --- |
|  | MCF-7 | **MDA-MB-231** | **Ishikawa** | **TEN** | **HEC1A** | **HEC1B** |
| ***ESR1*** | **++** | **−** | **+** | **−** | **−** | **−** |
| ESR2 | weak + | weak + | weak + | weak + | weak + | weak + |

Note: ++: strong expression; +: normal expression; weak +: weak expression and −: no expression

**Table S3. The Mean ± SD Ct values of *ESR2*** **and**

**corresponding *GAPDH* in each cell line**

| **Cell lines**    **Mean±SD**  **(Ct)**  **Genes** | **HEC1A** | **Ishikawa** | **TEN** | HEC1B | **MCF-7** | **MDA-MB - 231** |
| --- | --- | --- | --- | --- | --- | --- |
| ESR2 | 31.875±1.043 | 32.232±1.021 | 30.948±0.831 | 32.124±0.565 | 32.309±1.214 | 32.053±0.641 |
| GAPDH | 14.898±0.274 | 15.212±0.047 | 13.557±0.293 | 14.763±0.398 | 14.500±0.132 | 15.587±0.488 |

| **EC Grades** | **Number of samples** | **Age** | **Number of patients > 65 years** |
| --- | --- | --- | --- |
| Control | 4 | 45-49 | 0 |
| **EC-grade 1 (type 1)** | 8 | 45-87 | 7 |
| **EC-grade 2 (type 1)** | 5 | 60-85 | 4 |
| **EC-grade 3 (type 2)** | 8 | 58-85 | 6 |

Table S4. Summary of patient endometrial specimens

**Table S5**. **Signal pathway inhibitors (From Abcam)**

**and concentrations used in this study**

| **Names of Inhibitors** | **Signal Pathways (Molecules) to be Inhibited** | **Final Concentrations** |
| --- | --- | --- |
| BAY11-7082 (#ab141228) | NF-κB [IκBα kinase (IKK) inhibitor] | 2 µM |
| 6-Amino-4-(4-phenoxyphenylethylamino) quinazoline (QNZ, #ab141588) | NF-κB (Both NF-κB activation and TNF-α production inhibitor) | 5 nM |
| NVP-BEZ235 (#ab120882) | PI3K/Akt/mTOR (Dual PI3K and mTORC1/2 inhibitor) | 25 nM |
| LY294002 (#ab120243) | PI3K (All of the class I PI3K isoforms inhibitor) | 2 µM |
| Ruxolitinib (#ab141356) | JAK/STAT (JAK1/2 inhibitor) | 1 µM |
| SB202190 (#ab120639) | p38MAPK (A selective p38 MAP kinase inhibitor) | 5 µM |
| U0126 (#ab120241) | MAPK (A MEK1/2 inhibitor) | 2 µM |

**Figure Legends:**

**Figure S1.** **PD-L1 mRNA expression in the 4 EC and 2 BC cell lines** The PD-L1 transcriptional levels in the cell lines were examined by qRT-PCR and depicted as fold-changes following normalization with the internal control of the GAPDH gene, and relative to the calibrator HEC1A cell line. **A:** A comparison of the PD-L1 transcriptional levels in EC and BC cell lines. Exact p-values of Ishikawa versus (vs) MCF-7: p=0.0813; Ishikawa vs MDA-MB-231: p<0.0001 and MCF-7 vs MDA-MB-231: p<0.000. **B:** A comparison of the PD-L1 transcriptional levels within the cell lines of EC. Exact corresponding p-values in EC cell lines: HEC1A vs Ishikawa: p<0.0001; HEC1A vs TEN: p=0.0007; HEC1A vs HEC1B: p=0.2157; Ishikawa vs TEN: p=0.1029; Ishikawa vs HEC1B: p<0.0001 and TEN vs HEC1B: p<0.0001. Data are presented as mean ± SEM. One-way ANOVA was employed with multiple comparisons of Tukey for statistics. (n = 4). **p ≤ 0.01; ***p ≤ 0.001 and ****p ≤ 0.0001. ns: not significant.

**Figure S2. PD-L1** **mRNA expression in EC specimens** The PD-L1 transcriptional levels in EC specimens were examined by qRT-PCR and compared between non-EC (n = 4) and all EC samples (n = 21) (**A**); non-EC (n = 4) and EC type 1 groups (n = 13) (**B**) and non-EC (n = 4) and EC type 2 (n = 8) groups (**C**). Data are presented as mean ± SEM. Mann-Whitney non-parametric t-test was employed for statistical analyses. *p ≤ 0.05. ns: not significant.
